# Allicin Induces Thiol Stress in Bacteria through *S*-Allylmercapto Modification of Protein Cysteines*[Fn FN2]

**DOI:** 10.1074/jbc.M115.702308

**Published:** 2016-03-23

**Authors:** Alexandra Müller, Jakob Eller, Frank Albrecht, Pascal Prochnow, Katja Kuhlmann, Julia Elisabeth Bandow, Alan John Slusarenko, Lars Ingo Ole Leichert

**Affiliations:** From the ‡ Institute of Biochemistry and Pathobiochemistry–Microbial Biochemistry,; ¶ Angewandte Mikrobiologie, and; ‖ Medizinisches Proteom-Center, Ruhr University Bochum, 44780 Bochum, Germany and; § Department of Plant Physiology, Rheinisch-Westfälische Technische Hochschule Aachen University, 52056 Aachen, Germany

**Keywords:** antibiotic action, bacteria, disulfide, Escherichia coli (E. coli), thiol, S-allylmercapto modification, allicin, garlic (Allium sativum), redox proteomics

## Abstract

Allicin (diallyl thiosulfinate) from garlic is a highly potent natural antimicrobial substance. It inhibits growth of a variety of microorganisms, among them antibiotic-resistant strains. However, the precise mode of action of allicin is unknown. Here, we show that growth inhibition of *Escherichia coli* during allicin exposure coincides with a depletion of the glutathione pool and *S*-allylmercapto modification of proteins, resulting in overall decreased total sulfhydryl levels. This is accompanied by the induction of the oxidative and heat stress response. We identified and quantified the allicin-induced modification *S*-allylmercaptocysteine for a set of cytoplasmic proteins by using a combination of label-free mass spectrometry and differential isotope-coded affinity tag labeling of reduced and oxidized thiol residues. Activity of isocitrate lyase AceA, an *S*-allylmercapto-modified candidate protein, is largely inhibited by allicin treatment *in vivo*. Allicin-induced protein modifications trigger protein aggregation, which largely stabilizes RpoH and thereby induces the heat stress response. At sublethal concentrations, the heat stress response is crucial to overcome allicin stress. Our results indicate that the mode of action of allicin is a combination of a decrease of glutathione levels, unfolding stress, and inactivation of crucial metabolic enzymes through *S*-allylmercapto modification of cysteines.

## Introduction

The treatment of infectious diseases is and has been one of the main challenges faced by medicine. Prior to the discovery of the first antibiotics, treatment of infections mainly relied on herbal medication. A plant that frequently was considered as effective against a variety of infectious diseases is garlic (*Allium sativum*). It has traditionally been used in many cultures to treat a variety of human medical conditions, such as lung diseases, arthritis, and the common cold (1).

**FIGURE 1.**
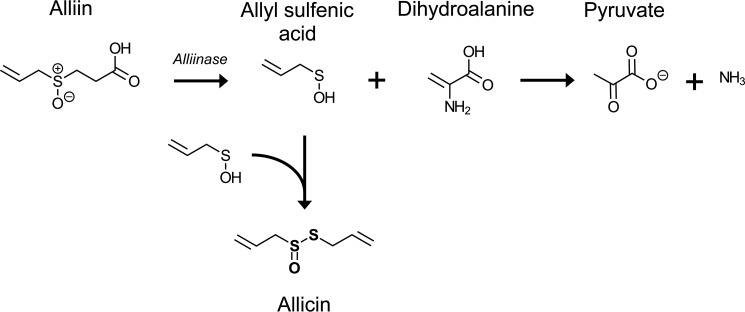
**Biosynthesis of allicin from alliin (adapted from Ref. 17).** Upon damage of garlic clove cells, alliin comes into contact with alliinase. Alliin is converted to allyl sulfenic acid and dihydroalanine. The latter is unstable and rapidly degrades to pyruvate and ammonia. Two molecules of allyl sulfenic acid spontaneously condense to allicin.

In 1944, diallyl thiosulfinate, the main antibacterially active ingredient of garlic, was isolated, and its chemical structure was described (2, 3). Based on its origin, diallyl thiosulfinate was named allicin. Allicin is a phytoanticipin released from the inactive odorless precursor alliin when garlic tissues are damaged by pathogens or pests. In intact *Allium* plant tissue, the inactive precursor alliin is present in the cytoplasm, separate from the C–S lyase alliinase, which is stored in the vacuoles (4). Upon mechanical damage by herbivores or due to infections by microorganisms, the vacuoles are disrupted, and alliin is converted to allyl sulfenic acid and dihydroalanine by alliinase (5, 6). Two molecules of allyl sulfenic acid spontaneously condense to allicin (Fig. 1).

The inhibitory effect of allicin against a variety of microorganisms, such as methicillin-resistant *Staphylococcus aureus*, *Streptococcus* spp., *Escherichia coli*, and *Salmonella enterica* serovar Typhimurium, as well as certain fungi and parasites has been studied extensively (2, 3, 7–12). Based on the results of these studies, allicin can be considered a broad range antimicrobial compound. The exact mode of antimicrobial action of allicin is not yet fully understood. As allicin is a hydrophobic substance that can easily pass membranes, it is likely that it can directly react with microbial molecules (13). *In vitro* studies using pure allicin demonstrated the inhibition of several enzymes, including thioredoxin reductase, papain, and alcohol dehydrogenase (14). As this inhibition could be rescued by the addition of reductants, such as DTT or 2-mercaptoethanol, the inhibition is likely caused by allicin-induced modification of cysteine thiols. Upon reaction of allicin with a sulfhydryl group, a disulfide is formed, resulting in an *S*-allylmercapto modification (15). This type of modification has been detected *in vitro* after incubation of allicin with proteins as well as with free cysteine and other thiol-containing molecules, such as glutathione (14–16). *S*-Allylmercapto-modified proteinogenic cysteines could, in principle, react further with free thiols present in the vicinity via a thiol-disulfide exchange reaction. This would result in the formation of intra- or intermolecular disulfide bonds between proteins and/or low molecular weight thiols, such as glutathione (17). Until now, detailed analyses of the global cellular consequences of allicin on the metabolism of microorganism as well as a global identification of the targets of allicin have not been performed. Feldberg *et al.* (9) observed an immediate inhibition of RNA synthesis in *S. enterica* Typhimurium after treatment with allicin, whereas protein and DNA synthesis was only delayed and partially inhibited. In yeast, allicin caused an increase in the cellular redox potential by oxidation of the glutathione pool, ultimately resulting in apoptosis (18).

In the present study, we provide a global analysis of allicin-induced cellular effects in the model organism *E. coli*. We show that pure allicin, synthesized from diallyl disulfide and hydrogen peroxide, inhibits growth of a broad range of pathogenic microorganisms, including Gram-positive and Gram-negative bacteria as well as fungi. Treatment of *E. coli* with a sublethal amount of allicin results in a significant reduction of total free sulfhydryl levels. This is, at least in part, due to a decrease of reduced glutathione. On a proteomic level, allicin treatment induces the heat stress and oxidative stress responses. Mass spectrometry revealed that allicin causes substantial *S*-allylmercaptocysteine modification of proteins *in vivo*. We were able to quantify the extent of modification for a subset of *S*-allylmercapto-modified proteins. Activity of isocitrate lyase, a major *S*-allylmercapto-modified candidate protein, was largely inhibited by allicin exposure. On a global scale, allicin-induced protein modifications resulted in enhanced protein aggregation, which in turn causes induction of the heat stress response through stabilization of the alternative heat shock σ factor σ^32^ (RpoH). Our results show that the antimicrobial effect of allicin coincides with a decrease in reduced glutathione levels that is not accompanied by a corresponding increase of glutathione disulfide, *S*-allylmercapto modification of proteins, and inhibition of metabolic enzymes.

## Experimental Procedures

### 

#### 

##### Synthesis of Allicin

Allicin was obtained by oxidation of diallyl disulfide with H_2_O_2_ as reported previously with the following modifications (18). 2 g of diallyl disulfide and 3 ml of 30% H_2_O_2_ were dissolved in 5 ml of glacial acetic acid prior to shaking at room temperature for 4 h. The reaction was stopped by addition of 25 ml of distilled water. Extraction was performed two times with 30 ml of diethyl ether prior to evaporation *in vacuo*. The remaining volume was resuspended in 25 ml of methanol (90%, v/v), and remaining diallyl disulfide was extracted several times with 10 ml of *n*-hexane until no diallyl disulfide was detectable by HPLC. The methanolic phase was removed by evaporation. The remaining volume was mixed with distilled water and extracted against diethyl ether. The ether phase was then dried with dry copper sulfate until no further discoloration from white to blue was observable. The ether was separated from the copper sulfate by filtration prior to evaporation. The resulting allicin had a purity of 95% as analyzed by HPLC.

##### Minimal Inhibitory Concentration Determination

*E. coli* K12 MG1655 was grown overnight in MOPS minimal medium (Teknova MOPS minimal medium kit; 1× MOPS mixture, 2% (w/v) glucose, 1.32 mm K_2_HPO_4_, 1 μg ml^−1^ thiamine) (19). 5 ml of MOPS medium were inoculated with 10^5^ cells of the overnight culture. Allicin (10 mg ml^−1^ stock) was added to each tube at different end concentrations (0–35 μg ml^−1^). Cultures were grown overnight at 37 °C on a roller drum. A non-inoculated culture with allicin (110 μg ml^−1^) and a culture inoculated with *E. coli* but without allicin served as controls. Minimal inhibitory concentration (MIC)^5^ determination of allicin against *E. coli* DSM 30083, *Acinetobacter baumannii* DSM 30007, *Pseudomonas aeruginosa* DSM 50071, *S. aureus* DSM 20231, *S. aureus* ATCC 43300 (methicillin-resistant *S. aureus*), and *Candida albicans* DSM 1386 was performed in a microtiter plate assay according to Clinical and Laboratory Standards Institute guidelines as described previously (20). *E. coli*, *A. baumannii*, and *S. aureus* were grown in Mueller Hinton broth, *P. aeruginosa* was grown in cation-adjusted Mueller Hinton II, and *C. albicans* was grown in Sabourand medium. Allicin was dissolved in dimethyl sulfoxide as a 1.25, 2.5, 5, or 10 mg ml^−1^ stock solution. Serial dilutions in culture media were prepared with the Tecan Freedom Evo 75 liquid handling work station (Tecan, Männedorf, Switzerland). Dilutions, starting from a 10 mg ml^−1^ stock solution, typically covered a range from 512 to 0.5 μg ml^−1^. Compound dilutions were inoculated with 5 × 10^5^ bacteria/ml from late exponential cultures grown in the same media. Assay volumes were 200 μl/well. Cells were incubated for 16–18 h at 37 °C. The lowest allicin concentration inhibiting visible bacterial growth was recorded as the MIC.

##### Stress Treatment of E. coli during Exponential Growth

*E. coli* K12 strains MG1655, MC4100, and MC4100_Δ*rpoH* were grown overnight in MOPS minimal medium or LB medium. 60 ml of MOPS medium or LB medium were inoculated to an *A*_600_ of 0.05, and strains were grown at 25 or 37 °C with shaking until an *A*_600_ of 0.5 was reached. Cultures were split into three 20-ml cultures and treated with 0.79 mm allicin, 1 mm diamide, or dimethyl sulfoxide (vehicle control). Growth of cultures was recorded for at least 700 min.

##### Determination of Sulfhydryl Contents by 5,5′-Dithiobis(2-nitrobenzoic acid) (DTNB) Assay

An *E. coli* MG1655 overnight culture was used to inoculate MOPS minimal medium (1:100). The culture was grown aerobically until an *A*_600_ of 0.4 was reached. The culture was split into three 15-ml cultures for stress treatment. An untreated culture served as a negative control. 0.79 mm allicin (128 μg ml^−1^) or 1 mm diamide was added to one of the remaining two cultures each. Cultures were incubated for 15 min. 5 ml of each culture were harvested by centrifugation (8,525 × *g*, 4 °C, 10 min). Cells were washed twice with 1 ml of PBS (137 mm NaCl, 2.7 mm KCl, 10 mm Na_2_HPO_4_, 2 mm KH_2_PO_4_, pH 7.4, stored anaerobically prior to use) and centrifuged (13,000 × *g*, 4 °C, 10 min). Cells were resuspended in lysis buffer (PBS with 6 mm guanidinium HCl, pH 7.4) prior to disruption at 4 °C by ultrasonication (VialTweeter ultrasonicator, Hielscher GmbH, Germany) (3 × 1 min). Cell debris was pelleted by centrifugation (13,000 × *g*, 4 °C, 15 min). The supernatant was transferred to a 3.5-ml QS-macro cuvette (10 mm) with a magnetic stir bar and mixed with 1 ml of lysis buffer. Extinction of the samples was monitored at 412 nm with a Jasco V-650 spectrophotometer equipped with the PSC-718 temperature-controlled cell holder (Jasco) at room temperature. 100 μl of a 3 mm dithiobis(2-nitrobenzoic acid) solution were added. Extinction was monitored until it reached saturation. Calculation of thiol concentration was performed using the extinction coefficient ϵ_412_ = 13,700 m^−1^ cm^−1^ for thio-2-nitrobenzoic acid (TNB). Cellular thiol concentrations were calculated based on a volume of *E. coli* cells of 6.7 × 10^−15^ liter and a cell density of *A*_600_ = 0.5 (equivalent to 1 × 10^8^ cells ml^−1^ culture) (21).

##### In Vitro Glutathione Determination

Reduced glutathione (GSH) and oxidized glutathione (GSSG) levels were determined as described previously with only minor changes (22). To test reactivity of allicin toward glutathione *in vitro*, 13 μm reduced and oxidized glutathione in KPE buffer (0.1 m potassium phosphate, pH 7.5, 5 mm EDTA) were incubated with 6.5 or 13 μm allicin in a total volume of 300 μl of KPE buffer for 15 or 60 min at room temperature. For the determination of total glutathione, 100 μl of the reaction mixture were added to a 1.5-ml QS-macro cuvette (10 mm) containing 45 μg ml^−1^ DTNB and 0.7 units ml^−1^ glutathione reductase (from *Saccharomyces cerevisiae*; Sigma-Aldrich) in a total volume of 880 μl of KPE buffer. After the addition of 45 μg ml^−1^ β-NADPH, the extinction of TNB was measured at 412 nm for 900 s in a V-650 spectrophotometer equipped with a PSC-718 temperature-controlled cell holder at room temperature. To block residual GSH, GSSG reaction mixtures were treated with 0.4% (v/v) 2-vinylpyridine for 60 min at room temperature prior to addition of 0.4% (v/v) triethanolamine. 110 μl of these reaction mixtures were used for glutathione determination. The initial slopes in Δ*E* min^−1^ (Δ*E*, change of absorbance at 412 nm) of GSH and GSSG standards with a concentration range between 26 and 0.1 μm in KPE buffer were used to calculate standard curves. Glutathione concentrations in the untreated control samples were set to 100%.

##### In Vivo Glutathione Determination

*E. coli* MG1655 was grown in MOPS minimal medium in a total volume of 200 ml until an *A*_600_ of 0.5 was reached. The culture was split into 50-ml cultures for stress treatment. After 15 min of incubation with 0.79 mm allicin, 1 mm diamide, or dimethyl sulfoxide (control), cells were harvested at 4,000 × *g* at 4 °C for 10 min. Cells were washed twice with KPE buffer prior to resuspension of pellets in 700 μl of KPE buffer. For deproteination, 300 μl of 10% (w/v) sulfosalicylic acid were added prior to disruption of cells by ultrasonication (3 × 1 min; VialTweeter ultrasonicator). Supernatants were collected after centrifugation (30 min, 13,000 × *g*, 4 °C). Sulfosalicylic acid concentrations were decreased to 1% by the addition of 3 volumes of KPE buffer. Measurements of total glutathione and GSSG were performed as described above. Cellular glutathione concentrations were calculated based on a volume of *E. coli* cells of 6.7 × 10^−15^ liter and a cell density of *A*_600_ = 0.5 (equivalent to 1 × 10^8^ cells ml^−1^ culture). GSH concentrations were calculated by subtraction of 2[GSSG] from total glutathione.

##### Two-dimensional Polyacrylamide Gel Electrophoresis (PAGE) Analysis

Radioactive labeling of newly synthesized proteins and subsequent separation of the cytosolic proteome by two-dimensional PAGE was performed as described previously (23). In short, 5 (radioactive gels) or 10 ml (non-radioactive gels) of an *E. coli* MG1655 culture in MOPS minimal medium were exposed to 0.79 mm allicin in the early exponential growth phase. After 10 min, cells were pulse-labeled with l-[^35^S]methionine for 5 min. Precursor incorporation was stopped by inhibition of protein biosynthesis by addition of 1 mg ml^−1^ chloramphenicol and an excess of non-radioactive methionine. Cells were harvested by centrifugation, washed three times with TE buffer (100 mm Tris, 1 mm EDTA, pH 7.5), and disrupted by ultrasonication. Methionine incorporation rates per protein content in the lysates were determined in duplicate for every replicate using PerkinElmer Life Sciences liquid scintillation analyzer Tri-Carb 2800 TR. Proteins from 1 μl of sample were TCA-precipitated on a Whatman filter disc by 20% (w/v) TCA for 20 min on ice. Filter discs were washed twice with 10% (w/v) TCA and then twice with 96% ethanol for 10 min each. Filter discs were dried and placed into a scintillation vial. 5 ml of scintillation solution (PerkinElmer Life Sciences Ultima Gold) were added prior to measurement of scintillation counts. Protein (55 μg for analytical; 300 μg for preparative gels) was loaded onto 24-cm immobilized pH gradient strips (pH 4–7; GE Healthcare) by passive rehydration for 18 h. Proteins were separated in a first dimension by isoelectric focusing and in a second dimension by sodium dodecyl sulfate (SDS)-PAGE using 12.5% acrylamide gels. Gels were stained with 0.003% ruthenium(II) tris(4,7-diphenyl-1,10-phenanthroline disulfonate) and imaged as described earlier (24). Analytical (radioactive) gels were dried and exposed to storage phosphor screens prior to scanning (24). Analytical gels of controls and allicin-treated samples were merged using Decodon Delta 2D image analysis software (Decodon, Greifswald, Germany). The 25 protein spots with the highest expression level on gels after allicin treatment were selected for protein identification. 21 of them were found on non-radioactive gels and were excised for mass spectrometric identification. Protein spots were identified by nano ultra-high pressure LC-electrospray ionization-MS/MS as reported previously using a Synapt G2-S HDMS mass spectrometer equipped with a lock spray source for electrospray ionization and a TOF detector (23). Mass spectrometry proteomics data for identification of spots from two-dimensional gels have been deposited to the ProteomeXchange Consortium (25) via the PRIDE partner repository with the data set identifier PXD003158.

##### Alkaline Phosphatase Activity Assay

The determination of PhoAΔ2–22 was performed as described previously (26). In brief, *E. coli* DHB4 harboring plasmid pAID135 were grown in MOPS minimal medium until an *A*_600_ of 0.2 was reached. Isopropyl β-d-1-thiogalactopyranoside was added to a final concentration of 5 mm to induce expression of leaderless alkaline phosphatase. At an *A*_600_ of 0.4, allicin (0.79 mm; 128 μg ml^−1^) or diamide (1 mm) was added to induce oxidative stress. After 15 min, 900 μl of cells were added to 100 μl of 1 m iodoacetamide prior to incubation on ice for 20 min. Cells were harvested and washed twice in 1 ml of prechilled washing buffer (50 mm NaCl, 10 mm NH_4_Cl, 10 mm MgCl_2_, 10 mm iodoacetamide, 40 mm MOPS-KOH, pH 7.3) prior to resuspension in 100 μl of lysis buffer (10 mm EDTA, 2 mg ml^−1^ lysozyme, 10 mm iodoacetamide, 20 mm Tris-HCl, pH 8.0) and 30-min incubation on ice. After lysing cells by three freeze-thaw cycles, 900 μl of resuspension buffer (10 mm MgCl_2_, 10 mm ZnCl_2_, 1 m Tris-HCl, pH 8.0) were added. Cell debris was cleared by centrifugation (11,000 × *g*, 10 min, 4 °C) prior to transfer of 900 μl of the supernatant to a quartz cuvette. Suspensions were stirred in a V-650 spectrophotometer equipped with a PSC-718 temperature-controlled cell holder for 10 min at 37 °C. The baseline extinction at 405 nm was recorded for 30 s prior to addition of 100 μl of *para*-nitrophenyl phosphate solution (0.4% (v/v) end concentration). Product formation was monitored for 2 h. Alkaline phosphatase activity in μm min^−1^ was calculated based on the initial velocity according to the following equation where ϵ_405_ *p-*nitrophenol = 18,000 m^−1^ cm^−1^ and Δ*E* is the change of absorbance at 405 nm min^−1^.

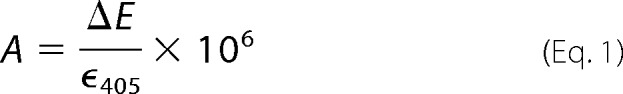
 The relative activity of the untreated control was set to 1.

##### Label-free Mass Spectrometric Analysis of the E. coli Proteome after Allicin Exposure

Culture growth, stress treatment, and cell disruption were performed as described for the DTNB assay. The supernatant was precipitated with 5 volumes of acetone overnight at −20 °C. After centrifugation (13,000 × *g*, 30 min, 4 °C) and washing with 5 volumes of acetone, the pellet was resuspended in ammonium bicarbonate buffer and digested with trypsin overnight (37 °C). Two individual replicates were analyzed for allicin treatment, and one sample was analyzed for control conditions and after diamide exposure. The LC-MS/MS consisted of an Ultimate 3000 RSLCnano system coupled online to an Orbitrap Elite Hybrid Ion Trap-Orbitrap mass spectrometer (Thermo Scientific). A total of 300 ng (in 15 μl) was preconcentrated on a trap column (Acclaim PepMap 100, 100 μm × 2 cm nanoViper, 5 μm, 30 μl min^−1^) prior to separation on an analytical column (Acclaim PepMap RSLC, C_18_, 2 μm, 100 Å, 5-μm inner diameter × 50 cm, nanoViper) using a gradient from 5 to 40% solvent B within 98 min (solvent A, 0.1% (v/v) formic acid; solvent B, 0.1% (v/v) formic acid, 84% (v/v) acetonitrile). The flow rate was set to 400 nl min^−1^ with a column oven temperature of 60 °C. Full scan data were acquired in the data-dependent mode. MS/MS analysis of the 20 most intense peaks was performed by collision-induced dissociation using the linear ion trap.

##### Data Analysis of S-Allylmercapto-modified Peptides

Data analysis was performed with MaxQuant Software Version 1.5.3.12 (27). For the identification, the *S*-allylmercapto modification of cysteines was added via the Andromeda configuration tab to the MaxQuant modification list (C_3_H_4_S; addition of 72.00337 Da) (27). Raw data files of both replicates were loaded into MaxQuant in parallel. “Oxidation (M)” and “*S*-allylmercapto” were set as variable modifications. Label-free quantification was selected. After data processing, the table evidence.txt was used for further analysis. Peptides that were found modified by *S*-allylmercapto (“*S*-allylmercapto”, “oxidation (M), *S*-allylmercapto,” and “2 *S*-allylmercapto”) were copied to a new excel sheet. The peptide list was further edited to display only those peptides that were identified in both replicates with a posterior error probability below 0.01. In the case where a peptide appeared in various modification states, the peptide with the lowest posterior error probability was selected.

##### OxICAT Labeling, Tryptic Digestion, and Peptide Enrichment

MOPS minimal medium was inoculated with 500 μl of an *E. coli* MG1655 overnight culture and cultivated until an *A*_600_ of 0.4 was reached. The main culture was split and stressed as described above. After 15 min of stress, 1.7 ml (corresponding to 100 μg of protein) of each culture were harvested and centrifuged (13,000 × *g*, 4 °C, 10 min). The supernatant was removed carefully, and pellets were washed twice with 1 ml of TE buffer (10 mm Tris-HCl, 1 mm EDTA, pH 8.5). Pellets were resuspended in 80 μl of denaturing alkylation buffer (6 m urea, 200 mm Tris, pH 8.5, 10 mm EDTA, 0.5% (w/v) SDS, freshly prepared and stored anaerobically prior to use). ICAT labeling was performed as described previously (28). Briefly, each sample was mixed with the content of one vial of light ICAT. Samples were incubated, protected from light, for 2 h at 37 °C with 300 rpm on a shaker. Samples were precipitated with 5 volumes of chilled acetone for 4 h or overnight. After washing and drying, the pellets were resuspended in 75 μl of denaturing alkylation buffer supplemented with 5 μl of 0.5 m tris(2-carboxyethyl)phosphine and incubated for 10 min. Subsequently, samples were mixed with the content of one vial of heavy ICAT and incubated for 2 h at 37 °C with 300 rpm on a shaker. One vial of lyophilized trypsin from the ICAT kit was restored in 200 μl of distilled water. Samples were resuspended in 80 μl of denaturing buffer from the ICAT kit and 20 μl of acetonitrile. 100 μl of the trypsin solution were added to each sample. Digests were performed for 16 h at 37 °C. Peptide purification by cation exchange and enrichment by affinity purification were carried out as described previously (28). For each condition (untreated, allicin-treated, and diamide-treated), three biological replicates were analyzed. LC-MS/MS analysis was performed as described above.

##### Data Analysis of OxICAT Samples

Raw data files of allicin-treated and control samples (three replicates each; parameter groups 1–6) were loaded to MaxQuant in parallel. ICAT-0 and ICAT-9 labels were selected in the group-specific parameters tab. “Oxidation (M)” was set as a variable modification. After data processing, the table peptides.txt was used for further data analysis.

##### Data Comparison of Label-free and OxICAT Experiments

The peptides from label-free analysis were matched to peptides from the OxICAT analysis. Peptides identified in both types of experiments were selected for OxICAT-based quantification. For this purpose, the degree of oxidation of a particular peptide in each individual experiment and replicate was calculated. Subsequently, the percentage of change between an untreated control and its allicin-treated counterpart was calculated.

Peptides were sorted by the average change of percentage points from all three replicates. Peptides with a change of percentage points above 10% in each of the replicates were considered consistent and reliable. Mass spectrometry proteomics data for identification of *S*-allylmercapto-modified cysteines (label-free) and quantification (OxICAT) have been deposited to the ProteomeXchange Consortium (25) via the PRIDE partner repository with the data set identifier PXD003063.

##### Isocitrate Lyase Activity Assay

Isocitrate lyase activity assays were performed as described earlier (29). In brief, *E. coli* MG1655 was grown as described for the DTNB assay. From each condition (untreated, allicin-treated, and diamide-treated), 4 ml of culture were harvested by centrifugation (13,000 × *g*, 10 min, 4 °C). Pellets were washed twice with imidazole buffer (50 mm imidazole, pH 6.8) prior to resuspension with 200 μl of imidazole buffer supplemented with 2 mg ml^−1^ lysozyme. Suspensions were incubated on ice for 30 min. Samples were disrupted by freeze-thaw lysis (three cycles of freezing in liquid nitrogen and thawing at room temperature; 30 s each). Cell debris was removed by centrifugation (13,000 × *g*, 10 min, 4 °C). Four 2-ml quartz cuvettes were filled with 750 μl of imidazole buffer, 150 μl of 10 mm EDTA, 150 μl of 50 mm MgCl_2_, 150 μl of 40 mm phenylhydrazine HCl, and 150 μl 10 mm dl-isocitric acid. The solutions were equilibrated to 30 °C using a V-650 spectrophotometer equipped with a PSC-718 temperature-controlled cell holder. Absorption was monitored at 324 nm until constant. 150 μl of isocitrate lyase (blank) and 150 μl of supernatants (the three test conditions) were added to the cuvettes. The increase in absorption was measured for 1,400 s. Activity was then calculated based on the extinction coefficient of phenylhydrazine glyoxylate, ϵ_324_ = 16,800 m^−1^ cm^−1^, and the velocity of the reaction.

##### Crude Extract Aggregation Assay

*E. coli* MC4100 was grown in 250 ml of LB medium to an *A*_600_ of 0.6. Cells were harvested by centrifugation at 4,000 × *g* for 20 min at 4 °C. The pellet was washed with 25 ml of washing buffer (50 mm HEPES-KOH, pH 8.0, 175 mm NaCl, 1 mm MgCl_2_) and resuspended in 1.25 ml of washing buffer. 100 μl of protease inhibitor mixture cOmplete, EDTA-free (one tablet/2 ml of distilled water; Roche Diagnostics) were added prior to cell disruption by ultrasonication (VialTweeter ultrasonicator; 3 × 1 min) at 4 °C. Lysates were cleared by centrifugation at 16,100 × *g* for 30 min at 4 °C. 40-μl aliquots were treated with allicin (1–100 mm) for 15 min at 30 °C. Samples were centrifuged for 30 min at 16,100 × *g* at 4 °C. Supernatants were mixed with 10 μl of 5× SDS sample buffer. Pellets were washed with 100 μl of washing buffer prior to resuspension in 100 μl of 1× SDS sample buffer. 2.5 μl of each sample were separated by denaturing SDS-PAGE.

##### In Vivo Degradation of RpoH

Turnover of RpoH was analyzed as described previously (26, 30). In brief, *E. coli* MG1655 harboring plasmid pEC5217 for the expression of RpoH was grown in LB medium at 30 °C until an *A*_600_ of 0.5 was reached. The culture was split into three 20-ml cultures prior to addition of 1 mm isopropyl β-d-1-thiogalactopyranoside. Diamide was added after 5 min to a final concentration of 1 mm, whereas allicin stress was started after 10 min by the addition of 0.79 mm allicin. After a total of 25 min, protein synthesis was stopped by the addition of 200 μg ml^−1^ chloramphenicol. 1-ml samples were collected after 0, 1, 3, 5, 7, 9, 11, 15, and 25 min and immediately frozen in liquid nitrogen. Samples were thawed on ice and centrifuged for 5 min at 4 °C and 16,100 × *g*. Pellets were resuspended in 40 μl of TE buffer (10 mm Tris, 1 mm EDTA, pH 8.0) and 10 μl of 5× SDS sample buffer. Samples were vortexed for 30 s and incubated at 95 °C for 5 min. Separation was performed by loading 10-μl samples on 4–12% Bis-Tris gels (Life Technologies) in MES running buffer. Separated proteins were transferred to nitrocellulose membranes using the iBlot system (Life Technologies) according to the manufacturer's instructions. Detection of RpoH was performed using polyclonal rabbit anti-RpoH antibodies and IRDye 680 LT goat anti-rabbit IgG (heavy + light; LI-COR Biosciences, Bad Homburg, Germany). Band intensities were quantified using ImageJ 1.49v (31).

## Results

### 

#### 

##### Allicin Inhibits Bacterial Growth

**FIGURE 2.**
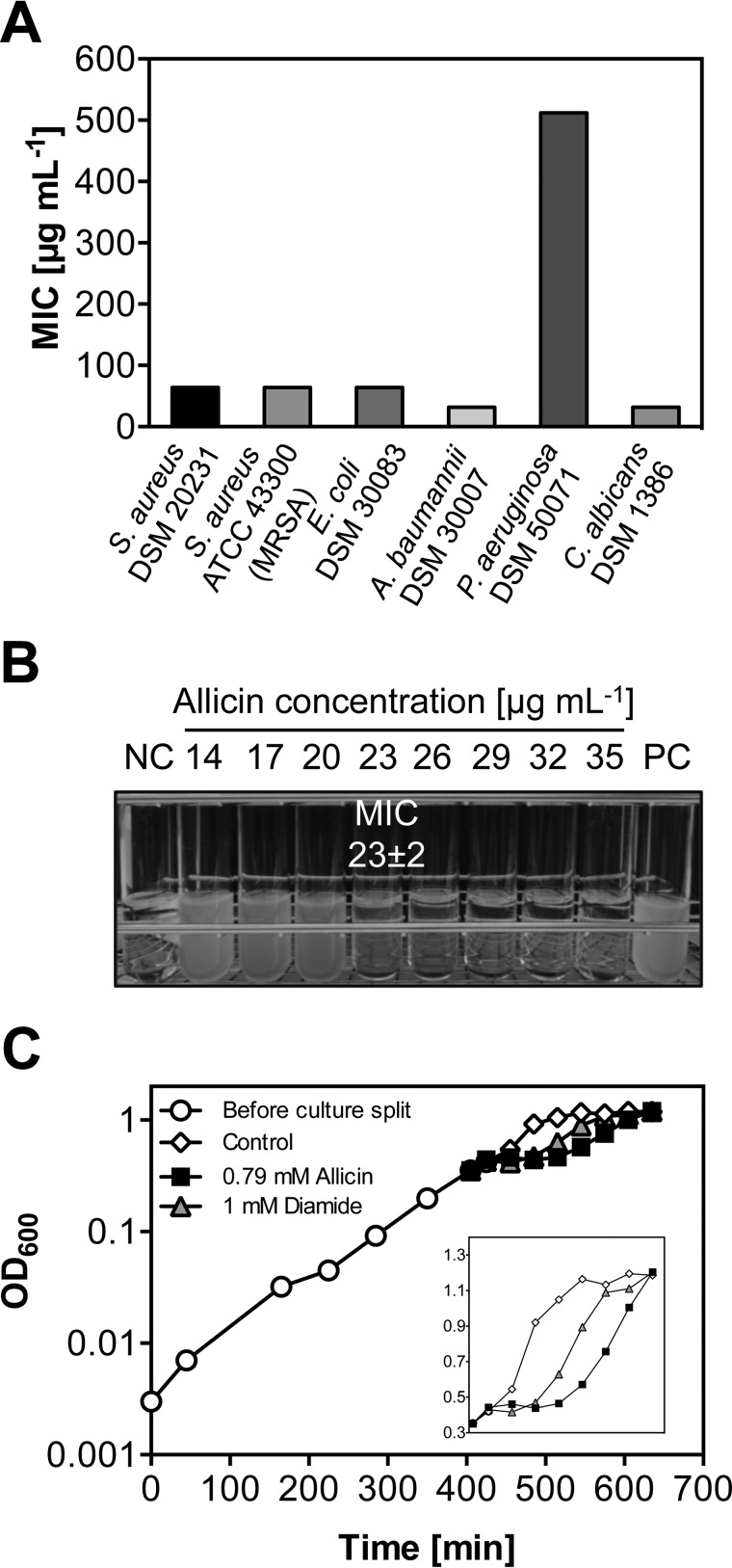
**Allicin inhibits growth of *E. coli* and other microorganisms.** *A*, MICs of allicin against pathogenic microorganisms tested according to Clinical and Laboratory Standards Institute standards in a microtiter plate-based assay using rich medium. *B*, a more precise determination of the minimal inhibitory concentration of allicin against *E. coli* MG1655 in MOPS minimal medium. Overnight cultures did not show visible growth at allicin concentrations above 23 μg ml^−1^. Negative control (*NC*), not inoculated; positive control (*PC*), without allicin. *C*, addition of 0.79 mm allicin (128 μg ml^−1^) or 1 mm diamide to midlogarithmic *E. coli* MG1655 (*black arrow*) causes a growth arrest. Cultures resumed growth after 60 and 90 min, respectively.

To analyze the effect of allicin on the growth of microorganisms, we determined the MIC according to Clinical and Laboratory Standards Institute standards on a set of pathogenic bacteria and fungi (Fig. 2*A*). The MIC of a substance represents the lowest concentration fully inhibiting visible growth. Allicin exhibited comparable MICs (32–64 μg ml^−1^) toward methicillin-resistant *S. aureus* ATCC 43300, the type strain *S. aureus* DSM 20231, *E. coli* DSM 30083, *A. baumannii* DSM 30007, and *C. albicans* DSM 1386. Growth of the type strain *P. aeruginosa* DSM 50071 was not affected by allicin concentrations lower than 512 μg ml^−1^. The Müller-Hinton medium used in standard MIC assays is a rich medium that contains thiols, which could inactivate allicin. We therefore determined the MIC of our laboratory strain *E. coli* MG1655 in a defined medium that lacks potentially inhibiting thiols (MOPS medium). A concentration of 23 μg ml^−1^ was sufficient to completely inhibit growth of *E. coli* in this medium (Fig. 2*B*).

We then determined allicin-induced inhibition of exponentially growing *E. coli*. Midlogarithmic cells showed a considerable growth inhibition at 128 μg ml^−1^ allicin (0.79 mm), which is approximately 6 times higher than the concentration needed for growth inhibition of the significantly smaller number of cells used in MIC tests (Fig. 2*C*). The disulfide stress inducer diamide served as a positive control for growth inhibition (26, 32, 33). Addition of both compounds led to an immediate growth arrest. After ∼60 min of diamide treatment and 90 min of allicin treatment, the cultures resumed growth and reached the same final optical densities as the control cultures. These results show that, although allicin seems to be slightly more inhibitory when compared with diamide, cells are able to overcome the stress induced by both compounds at the levels used. This experiment provided a suitable basis for the in-depth analysis of the cellular consequences of allicin exposure at sublethal levels.

##### Allicin Reduces Cellular Thiol Levels

**FIGURE 3.**
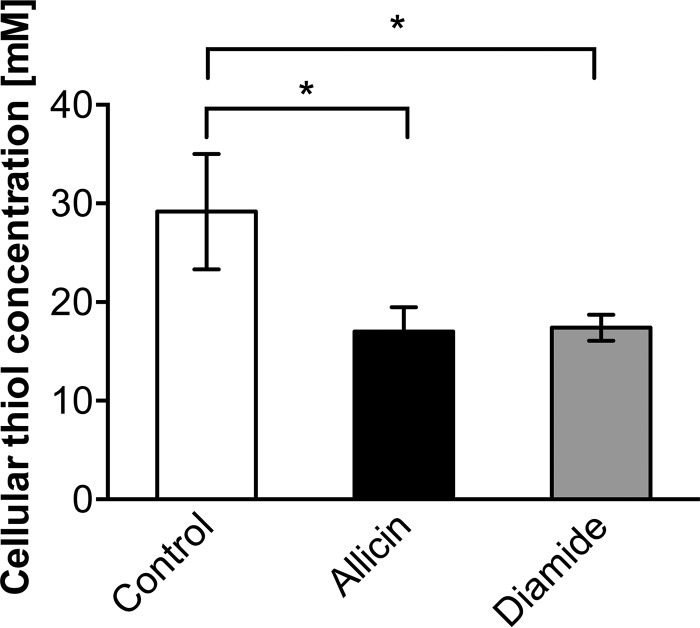
**Allicin induces a decline of sulfhydryl levels.** Treatment of cells with 0.79 mm allicin or 1 mm diamide leads to a significant decrease of total cellular free thiols as measured with DTNB. Values are the means and S.D. (*error bars*) from three biological replicates. *Asterisks* indicate a *p* value below 0.05 as determined by an unpaired Student's *t* test.

Four main groups of thiol-containing molecules contribute to the total cellular thiol content: glutathione, free cysteine, other low molecular weight thiols, and cysteine-containing proteins. As allicin is considered a thiol-reactive compound, we directly measured the total sulfhydryl concentrations in crude extracts of cells treated with allicin. The quantification of free thiol levels was carried out using DTNB (Ellman's reagent). DTNB reacts with free sulfhydryls and forms a mixed disulfide as well as TNB. The resulting TNB concentration can be determined spectrophotometrically at 412 nm and is directly proportional to the thiol concentration in the sample. The sulfhydryl concentrations of cells treated with 0.79 mm allicin or 1 mm diamide decreased by ∼40% when compared with the untreated control (Fig. 3). This corresponded to a drop of free thiols by 12 mm within the cell; thus allicin reacts with free thiols with high efficiency.

##### Allicin Reacts with Glutathione in Vitro and in Vivo

**FIGURE 4.**
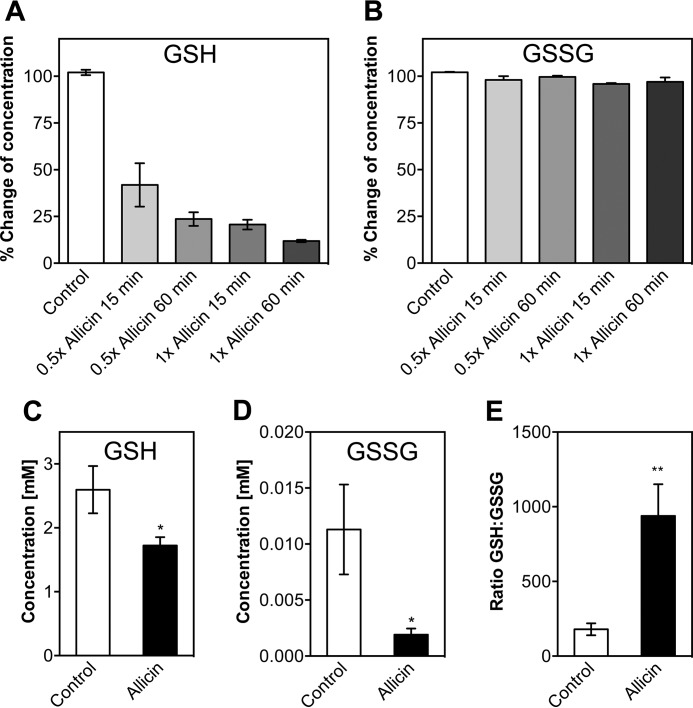
**Allicin reacts with reduced glutathione *in vitro* and *in vivo*.** Allicin was incubated with GSH (*A*) or GSSG (*B*) in a 0.5- or 1-fold molar ratio for 15 or 60 min. Subsequently, GSH and GSSG levels were measured and compared with standard curves. Concentrations of the untreated controls were set to 100%. Levels of reduced (*C*) and oxidized glutathione (*D*) were measured in *E. coli* cells after treatment with allicin for 15 min. *E*, the GSH:GSSG ratio is increased upon allicin stress. Values are the means and S.D. (*error bars*) from three biological replicates. *Asterisks* indicate a *p* value below 0.05 (*) or 0.01 (**) as determined by an unpaired Student's *t* test.

Glutathione is the most important low molecular weight antioxidant in the cell. With its thiol group, GSH can react with a variety of reactive species. This often leads to the formation of GSSG. Typically, the GSH to GSSG ratio in the cell is kept high by the enzyme glutathione oxidoreductase, and the concentration of GSH exceeds the concentration of GSSG by a factor of >100 (34). We asked whether the observed decrease in total thiol levels during allicin stress is due to reaction of allicin with GSH (Fig. 4). First, we tested whether allicin reacts with reduced GSH *in vitro*. A decrease to ∼40% of the initial total glutathione level was observed at molar ratios of allicin to GSH of 0.5 after 15 min of incubation. GSH levels dropped more with prolonged incubation (60 min) and at higher molar ratios (Fig. 4*A*). GSSG was not affected by allicin (Fig. 4*B*). The assay we used is able to determine the total glutathione concentration (*i.e.* [GSH] + 2[GSSG]). Our results indicate that allicin is indeed reacting with free GSH, but this does not result in the formation of GSSG. Instead, the reaction product cannot be reduced by glutathione oxidoreductase. It has been reported previously that the reaction of GSH with allicin leads to the formation of *S*-allylmercaptoglutathione (GSSA) (16). The observed decrease of total glutathione in the presence of allicin suggests that GSSA was not effectively converted to GSSG by a reaction with GSH and was not transformed to GSH and allylmercaptan by the action of glutathione oxidoreductase present in the assay.

*In vivo* analysis of glutathione levels in *E. coli* cells during the exponential growth phase revealed that GSH levels drop significantly under allicin stress, similar to our *in vitro* observation (Fig. 4*C*). This drop in GSH is not accompanied by a rise in GSSG levels. Quite the contrary, GSSG levels in allicin-stressed cells decreased when compared with control cells (Fig. 4*D*). Thus, although the overall glutathione level drops, the ratio of the GSH/GSSG couple in allicin-stressed cultures is significantly increased about 5-fold (Fig. 4*E*). It seems that, once it reacts with allicin, glutathione does not reenter, or only slowly reenters, the GSH pool.

##### Allicin Induces Heat Shock and Oxidative Stress Response

**FIGURE 5.**
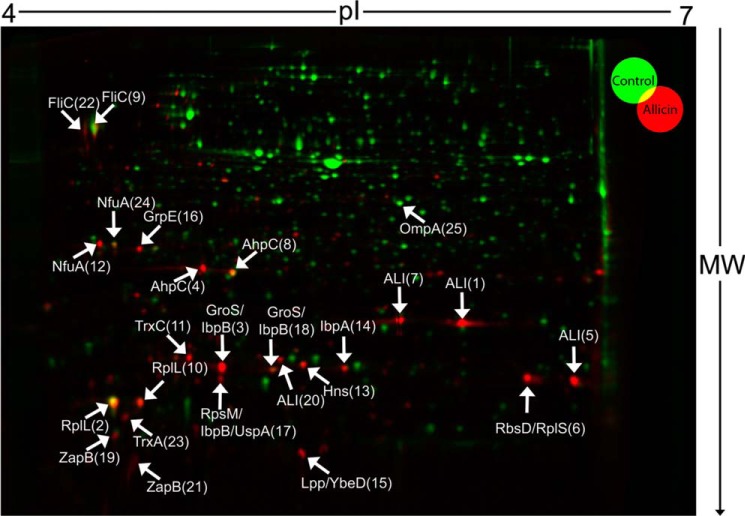
**Allicin treatment induces heat shock and oxidative stress response in *E. coli*.** Newly synthesized proteins of untreated (*green*) and allicin-treated (*red*) *E. coli* cultures were analyzed by [^35^S]methionine labeling prior to separation by two-dimensional PAGE and detection of spots using a phosphor screen. [^35^S]Methionine incorporation was found to be 44% of the control in the allicin-treated culture, suggesting a decreased protein synthesis upon allicin treatment. An overlay of gel images was performed to identify proteins expressed in response to allicin treatment. The 25 spots with the highest synthesis rates in allicin-treated *E. coli* were cut from non-radioactive gels, trypsin-digested, and analyzed by mass spectrometry. The *numbers* behind protein names represent spot numbers in Table 1. *ALI* indicates allicin-induced spots that could not be identified by mass spectrometry. Shown are representative gels from three individual biological replicates.

To explore the cellular consequences of the drop in free cellular thiols and glutathione, we performed proteomic profiling using two-dimensional gel electrophoresis (Fig. 5). Cells were treated with allicin, and newly synthesized proteins were labeled with l-[^35^S]methionine. The [^35^S]methionine incorporation rates indicated a moderate decrease of protein synthesis after allicin exposure to 44% of the synthesis rate of untreated cells. Newly synthesized proteins could then be quantified by densitometry of radioactive spots after two-dimensional PAGE (Fig. 5). Allicin treatment led to a drastic change in the protein synthesis profile (Fig. 5). We then identified the 25 protein spots with the highest synthesis rates in allicin-treated cells. 21 of these spots could be identified by mass spectrometry from non-radioactive gels (Fig. 5, Table 1, and supplemental Table 1). From 16 spots, a single protein was identified, whereas the remaining five spots represented two or more proteins. Remarkably, many of the identified proteins, such as IbpA and AhpC, belong to the σ^32^ or the OxyR regulon. The heat shock σ factor σ^32^ together with RNA polymerase regulates the expression of genes crucial for the adaptation to heat stress, whereas OxyR is an important transcriptional regulator for the oxidative stress response in *E. coli* (35, 36). We reported previously that disulfide stress induces the heat stress response in *E. coli* (26). In this earlier study, disulfide stress was induced chemically by diamide and genetically by a deletion of *trxB* and *gor*, which are the ultimate NADPH-dependent reductases of the thioredoxin and glutathione/glutaredoxin systems, respectively. The proteomic results presented here are strikingly similar to our findings in our previous study; we thus hypothesized that allicin may induce disulfide stress similarly to diamide.

**TABLE 1:** **The 25 proteins with the highest synthesis rate in allicin-treated *E. coli* identified from two-dimensional gels (see Fig. 5)** NA, not applicable.

Spot ID	Protein (EG number) (53)	Protein function (53)	Theoretical molecular mass	Theoretical pI (pH)
			*Da*	
1	ALI1 (NA)	Unidentified allicin-induced protein 1	NA	NA
2	RplL (EG10873)	50S ribosomal subunit protein L7/L12	12,287	4.3989
3	GroS (EG10600)	Chaperonin Cpn10	10,380	4.9585
	IbpB (EG11535)	Chaperone, heat-inducible protein of HSP20 family	16,083	5.02
4	AhpC (EG11384)	Alkyl hydroperoxide reductase, subunit C	20,748	4.8486
5	ALI5 (NA)	Unidentified allicin-induced protein 5	NA	NA
6	RbsD (EG10817)	d-Ribose pyranase	15,282	5.9326
	RplS (EG10880)	50S ribosomal subunit protein L19	13,125	11.0625
7	ALI7 (NA)	Unidentified allicin-induced protein 7	NA	NA
8	AhpC (EG11384)	Alkyl hydroperoxide reductase, subunit C	20,748	4.8486
9	FliC (EG10321)	Flagellin, structural gene	51,264	4.3008
10	RplL (EG10873)	50S ribosomal subunit protein L7/L12	12,287	4.3989
11	TrxC (EG11887)	Thioredoxin 2, zinc binding	15,544	4.8164
12	NfuA (EG12935)	Fe/S biogenesis protein	20,984	4.3271
13	Hns (EG10457)	DNA-binding global regulator H-NS	15,530	5.2412
14	IbpA (EG11534)	Chaperone, heat-inducible protein of HSP20 family	15,764	5.4785
15	Lpp (EG10544)	Murein lipoprotein	8,318	9.7734
	YbeD (EG11592)	UPF0250 family protein	9,821	5.4053
16	GrpE (EG10416)	Nucleotide exchange factor for the DnaKJ chaperone	21,784	4.4839
17	RpsM (EG10912)	30S ribosomal subunit protein S13	13,091	11.2163
	IbpB (EG11535)	Chaperone, heat-inducible protein of HSP20 family	16,083	5.02
	UspA (EG11390)	Global regulatory gene for stress response	16,056	4.9219
18	GroS (EG10600)	Chaperonin Cpn10	10,380	4.9585
	IbpB (EG11535)	Chaperone, heat-inducible protein of HSP20 family	16,083	5.02
19	ZapB (EG11878)	FtsZ stabilizer, coiled coil protein	9,628	4.4883
20	ALI20 (NA)	Unidentified allicin-induced protein 20	NA	NA
21	ZapB (EG11878)	FtsZ stabilizer, coiled coil protein	9,628	4.4883
22	FliC (EG10321)	Flagellin, structural gene	51,264	4.3008
23	TrxA (EG11031)	Thioredoxin 1	11,799	4.4751
24	NfuA (EG12935)	Fe/S biogenesis protein	20,984	4.3271
25	OmpA (EG10669)	Outer membrane protein A	37,177	5.9678

##### Allicin Did Not Induce Protein Disulfide Bond Formation in Vivo

We thus tested whether allicin causes non-native protein disulfide bond formation in the cytoplasm. For our experiment, we measured the activity of leaderless alkaline phosphatase PhoAΔ2–22 (37). Alkaline phosphatase activity depends on the formation of two disulfide bonds that contribute to the native protein fold. Wild-type alkaline phosphatase is secreted into the periplasm where oxidative conditions lead to disulfide bond formation (38). Leaderless alkaline phosphatase, however, lacks the signal sequence for the export into the periplasm and thus is inactive in the reducing environment of the cytoplasm under non-stress conditions. Oxidative stressors, such as diamide and genetically induced disulfide stress, however, can activate PhoAΔ2–22 despite its localization in the cytoplasm (26).

**FIGURE 6.**
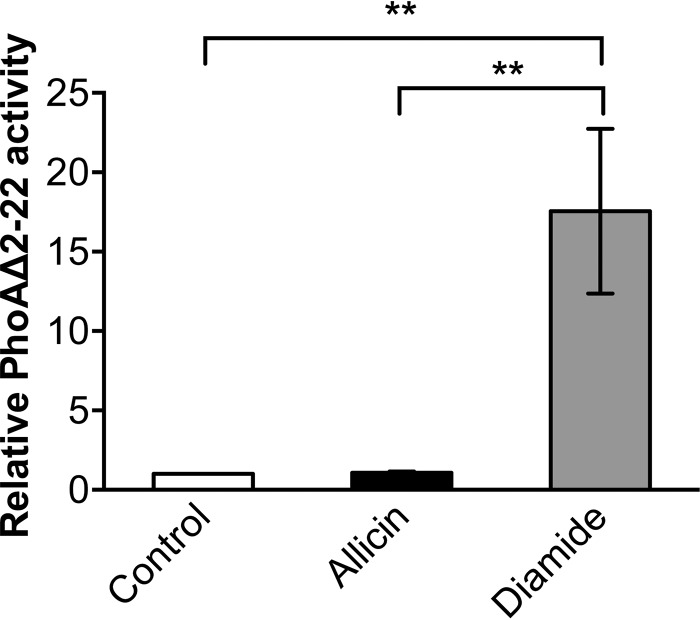
**Allicin does not induce the formation of disulfide bonds in PhoAΔ2–22.** Measurement of the activity of leaderless alkaline phosphatase shows that allicin in contrast to diamide does not readily induce cystine bond formation in this protein. The *para*-nitrophenyl phosphate conversion rate of the control was set to 1. Values are the means and S.D. (*error bars*) from three biological replicates. *Double asterisks* indicate a *p* value below 0.01 as determined by an unpaired Student's *t* test.

However, lysates of cells treated with allicin did not show an activity level of PhoAΔ2–22 above the levels of untreated cells (Fig. 6). In contrast, 1 mm diamide, a potent inducer of protein disulfide bond formation, significantly activated PhoAΔ2–22. This result shows that although allicin and diamide both decrease total sulfhydryl levels of the cell as well as levels of reduced and oxidized glutathione and induce the heat stress response, the underlying mechanisms differ significantly. Formation of cystine disulfide bonds in cytoplasmic proteins does not seem to be as common in allicin-treated cells as it is in cells that experience disulfide stress caused by diamide or knock-out of thiol-disulfide oxidoreductase systems.

##### Allicin Exposure Leads to S-Allylmercapto Modification of Proteins in Vivo

Our *in vitro* and *in vivo* data on the reaction of GSH with allicin suggested that *S*-allylmercapto-modified glutathione is a major product of this reaction. It thus seemed likely to us that proteins are also *S*-allylmercapto-modified upon treatment of cells with allicin. To analyze whether the proposed *S*-allylmercapto modification is indeed a stable end product of protein modification by allicin *in vivo*, lysates of allicin-treated *E. coli* cells were prepared under non-reducing conditions. Lysates of untreated cells served as a negative control, and chemically induced disulfide stress in diamide-treated cells served as a further control. After tryptic digestion, samples were directly analyzed by mass spectrometry in a label-free approach. We then searched for the *S*-allylmercapto modification at cysteine residues. A total of 90 allylmercapto-modified peptides were identified in our experiments with high confidence. These 90 peptides belong to a total of 73 individual proteins (supplemental Table 2). In contrast to allicin, no *S*-allylmercapto-modified peptides were identified in the control and the diamide-treated sample.

##### Individual Cysteines Were Up to 90% S-Allylmercapto-modified

To quantify the extent of *S*-allylmercapto modification, we then performed differential OxICAT labeling of cysteines from the *E. coli* proteome after allicin exposure (28). This differential labeling method is based on the isotopically different but chemically identical labels ICAT-^12^C (light) and ICAT-^13^C (heavy). The light version of the probe was used to label and thereby block all initially reduced cysteines. After treatment with a strong reductant, newly accessible cysteine thiols were labeled with the heavy probe. After digestion, peptide purification, and mass spectrometry, the ratio of light and heavy fractions of a peptide could be calculated. This ratio directly reflects the *in vivo* ratio of thiol modification of protein cysteines.

Of the cysteines we initially identified as *S*-allylmercapto-modified, 12 showed a rate of modification consistently higher than 10%, reaching as high as 90% (Table 2 and supplemental Table 3). The respective proteins are high abundance proteins mostly involved in the primary metabolism of *E. coli*, suggesting that allicin targets cytosolic proteins indiscriminately. Interestingly, our MS data suggest that in some of these proteins, which possess cysteines that are important for their activity, allicin predominantly modified cysteines that have not been implied in catalysis or redox regulation; *e.g.* 25% of cysteine 289 of glyceraldehyde-3-phosphate dehydrogenase (GapA), an important glycolytic enzyme, was modified. GapA is known to be subject to redox regulation of the enzyme upon oxidative stress (28, 39). Typically, this regulation occurs directly at the active site cysteine of this enzyme. Strikingly, allicin, unlike other oxidants, such as HOCl, did not seem to modify the catalytic cysteine but instead targets an unrelated cysteine. Similarly, GrxC, a disulfide reductase of *E. coli*, was modified at a single cysteine that is not part of the catalytic C*XX*C motif (40, 41). In isocitrate lyase (AceA), we only found Cys-244 to be *S*-allylmercapto-modified, whereas peptides containing Cys-195, an important residue for catalytic activity (42), were not detected in all three replicates and changed to a much lesser extent.

**TABLE 2:** **Cysteine-containing peptides in the *E. coli* proteome that are S-allylmercapto-modified and show a high quantity of modification in allicin-treated cells** For more detailed information, see supplemental Tables 2 and 3.

Peptide	Protein (EG Number) (53)	Protein function (53)	Average change of oxidation	Standard deviation change of oxidation	Oxidation control	Oxidation allicin-treated	Accessible surface area*^a^*
			%	%	%	%	%
S**C**VEVARLPK	RidA (EG12524)	Enamine/imine deaminase, reaction intermediate detoxification	88.9	1.4	1.5	90.4	16.32
TDADAADLITSD**C**DPYDSEFITGERTSEGFFR	AceA (EG10022)	Isocitrate lyase	51.0	14.0	1.2	52.2	20.12
**C**TQELLFGKGSALINDKR	AspC (EG10096)	Aspartate aminotransferase, AspAT	47.0	13.7	0.7	47.7	13.08
LLVDA**C**YSPVER	RpoA (EG10893)	RNA polymerase, α subunit	41.4	6.4	1.7	43.1	51.09
AAENNPELAAFIDE**C**RNTK	LeuS (EG10532)	Leucine-tRNA ligase	36.1	12.6	1.8	37.9	13.06
ILNTSSVIPVDGL**C**VR	Asd (EG10088)	Aspartate-semialdehyde dehydrogenase	31.1	14.4	2.1	33.2	10.57
VAIDAINAAGAPH**C**FLSVTK	AroG (EG10079)	3-Deoxy-d-arabinoheptulosonate-7-phosphate synthase	25.5	9.9	2.6	28.1	21.45
GVLGYTEDDVVSTDFNGEV**C**TSVFDAK	GapA (EG10367)	Glyceraldehyde-3-phosphate dehydrogenase A	24.9	8.4	0.9	25.8	16.83
TTVPQIFIDAQHIGG**C**DDLYALDAR	GrxC (EG12294)	Glutaredoxin 3	24.8	2.8	2.5	27.3	13.20
GTVVDIPAL**C**DALASK	SerA (EG10944)	d-3-Phosphoglycerate dehydrogenase	23.9	11.3	1.8	25.8	6.61
VGAGPFPTELFDETGEFL**C**K	PurA (EG10790)	Adenylosuccinate synthase, purine synthesis	18.8	8.2	0.9	19.7	7.72
LLTT**C**NIPVPSDVR	Pgk (EG10703)	Phosphoglycerate kinase	16.3	7.7	1.0	17.3	19.19

*^a^* As calculated by POPS (43) from Protein Data Bank-deposited structure data.

##### Allicin Reduces Isocitrate Lyase Activity in Cell Lysates of E. coli

**FIGURE 7.**
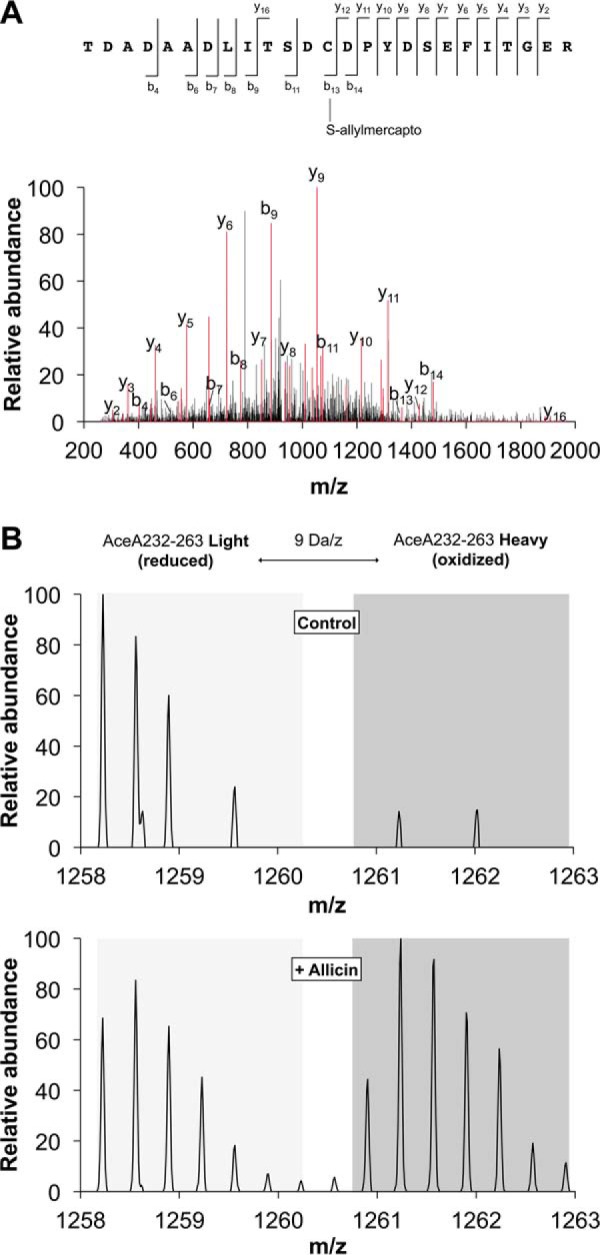
**Allicin leads to substantial *S*-allylmercapto modification of cellular proteins.** *A*, MS/MS annotation of peptide AceA232–256 identifies the *S*-allylmercapto modification of cysteine 244 induced by allicin treatment. The b- and y-ion series are marked in *red*. The total mass is consistent with an *S*-allylmercapto modification of cysteine 244 and fragments b_13_, b_14_, and y_16_ provide direct evidence. *B*, OxICAT quantification of AceA232–263 containing the *S*-allylmercapto-modified cysteine 244. MS spectra of this peptide show increased labeling with the heavy ICAT label after allicin treatment, corresponding to a higher level of oxidation of the respective cysteine. Representative spectra from one of the three biological replicates are displayed.

**FIGURE 8.**
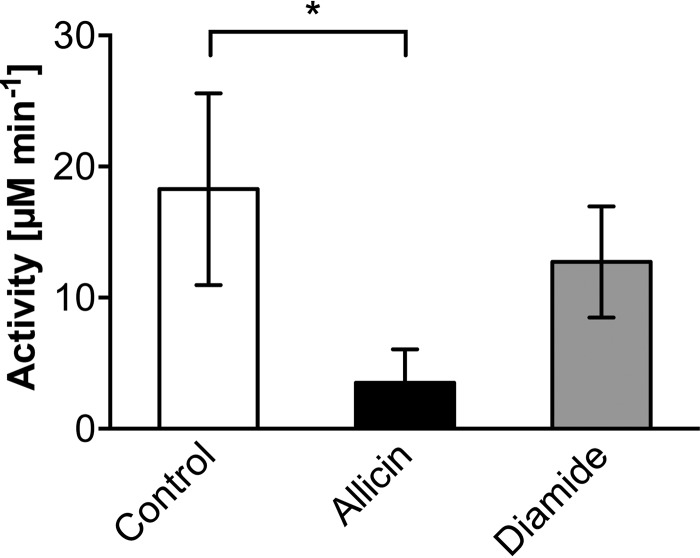
**Isocitrate lyase activity is reduced in lysates of *E. coli* cells treated with allicin.** Although diamide treatment (1 mm) had a minor effect on AceA activity, treatment with 0.79 mm allicin led to significant enzyme inhibition. Values are the means and S.D. (*error bars*) from three biological replicates. The *asterisk* indicates a *p* value below 0.05 as determined by an unpaired Student's *t* test.

To further explore whether these modifications lead to an allosteric inactivation of cellular enzymes, we looked directly at the activity of isocitrate lyase AceA. Isocitrate lyase plays a crucial role in the glyoxylate cycle, which bypasses the decarboxylation steps of the tricarboxylic acid cycle (26). We tested AceA activity in lysates of *E. coli* cells after allicin and diamide treatment. This candidate was chosen for two reasons. First, in our OxICAT analysis, we found that Cys-244 of this enzyme, a residue not involved in catalysis, was *S*-allylmercapto-modified (Fig. 7*A* and Table 2). This cysteine showed an average change in the oxidation state of 51%, which makes it the second most oxidized candidate identified in our analysis (Fig. 7*B* and Table 2). Second, activity assays for isocitrate lyase are well established and can be performed with cell extracts. AceA activity was significantly diminished after treatment with allicin (Fig. 8). Diamide treatment, which was performed as a control, also had an inhibitory effect when compared with control lysates. However, activity was significantly higher and twice as high when compared with allicin treatment. This result indicates that enzyme activity, at least of AceA, is inhibited by allosteric modification in allicin-treated cells.

##### Allicin Causes Protein Aggregation

**FIGURE 9.**
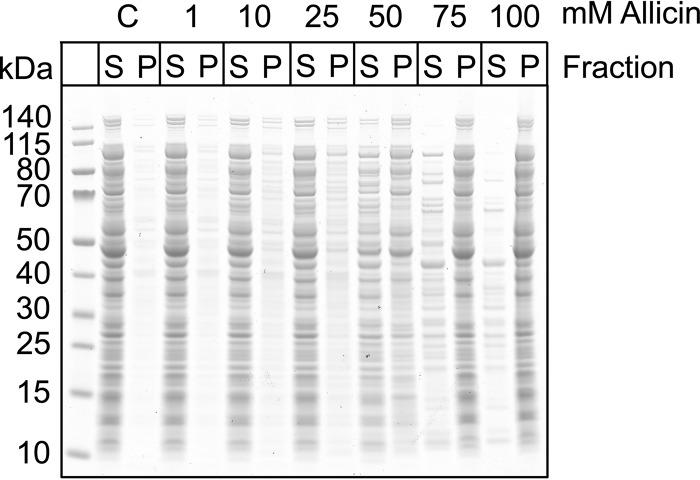
**Allicin induces protein aggregation.** Increasing concentrations of allicin were applied to crude extracts from *E. coli* for 15 min at room temperature. Proteins were separated into soluble (*S*) and insoluble fractions (*P*) by centrifugation prior to SDS-PAGE. An increased amount of proteins in the pellet fraction indicates aggregation. *C*, control.

Our data indicate that allicin induces *S*-allylmercapto modification of proteins and that this modification can inhibit protein activity. This observation could be explained by unfolding stress caused by *S*-allylmercapto modification. Additionally, protein unfolding and aggregation would be a potent inducer of the heat shock response, which could explain the strong up-regulation of heat shock proteins on our two-dimensional gels. To test this hypothesis, we performed aggregation assays with increasing allicin concentrations using crude extracts of *E. coli* MC4100. This strain was used previously to analyze protein aggregation upon diamide stress (26). Allicin caused protein aggregation in a concentration-dependent manner that was already visible at 1 mm allicin (Fig. 9). At concentrations higher than 50 mm, the majority of proteins aggregated and could be found in the pellet fraction. Diamide applied at the same concentrations did not cause aggregation in wild-type *E. coli* crude extracts in our previous study (26). This finding suggests that allicin-induced modifications affect proper folding and structural integrity of cellular proteins more than disulfide stress caused by non-native cystine bond formation.

##### Allicin Exposure Leads to RpoH Stabilization

**FIGURE 10.**
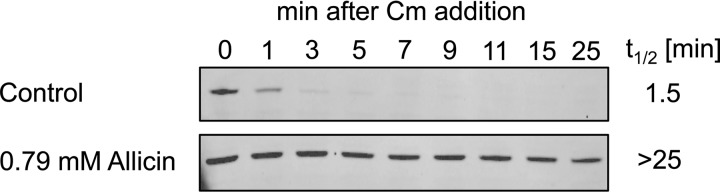
**RpoH is strongly stabilized upon allicin stress.** After 15 min of allicin stress, protein synthesis was blocked by the addition of chloramphenicol (*Cm*), and samples were taken after the time points indicated. RpoH was visualized by SDS-PAGE and Western blotting. Half-lives (*t*_½_) were calculated from band intensities after analysis with ImageJ 1.49v (31).

In *E. coli*, the induction of the heat stress response depends on the heat shock σ factor σ^32^ (RpoH) (36). Under non-stress conditions, RpoH is bound by DnaK/J and rapidly targeted to degradation by FtsH. Under heat stress, however, DnaK/J bind to denaturing proteins to protect them from aggregation or to target them to proteolysis. RpoH thus is stabilized and induces the expression of chaperones and other heat shock proteins in concert with RNA polymerase (36). Induction of the heat stress response upon allicin stress may thus be due to increased stability of RpoH caused by protein unfolding and aggregation. We therefore tested RpoH stability in cells exposed to allicin. RpoH levels remained stable during 25 min of analysis (Fig. 10). In contrast, the RpoH half-life was very short under control conditions. Thus, like diamide (26), allicin exposure leads to a strong stabilization of RpoH, which results in the induction of the heat shock response.

##### RpoH Is Crucial to Overcome Allicin Stress

**FIGURE 11.**
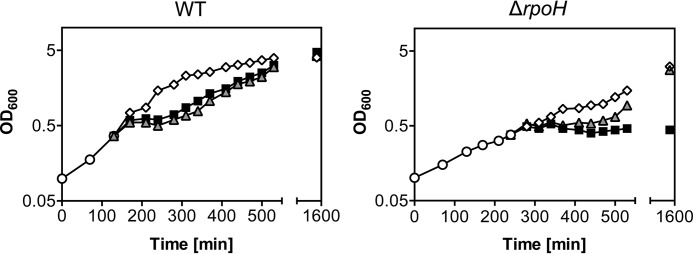
***E. coli* lacking RpoH does not recover from allicin stress.** *E. coli* strains (BB7222 and BB7224; MC4100 and MC4100 Δ*rpoH* (26)) were grown in LB medium at 25 °C (○). At an *A*_600_ of 0.5, cultures were split and treated with 0.79 mm allicin (■), 1 mm diamide (▴), or dimethyl sulfoxide (control) (♢). Growth was controlled for ∼25 h.

At sublethal concentrations (0.79 mm), *E. coli* can overcome allicin stress and resume growth after ∼90 min (Fig. 2*C*). One explanation could be the increased expression of chaperones and proteins belonging to the oxidative stress response, which would ultimately remove allicin-damaged proteins from the cell. We thus asked whether chaperones and other heat shock proteins synthesized during allicin exposure are essential for this recovery. We tested recovery of an *E. coli rpoH* mutant from allicin stress. This mutant is not able to induce the heat stress response. Growth of this mutant strain is very slow, and cells can only grow at moderate temperatures (25 °C). Although this strain could recover from diamide stress after a long phase of growth arrest, it did not resume growth after allicin exposure (Fig. 11). It thus seems that the induction of the heat stress response accompanied by the expression of chaperones and other heat stress proteins is essential upon allicin stress.

## Discussion

Allicin from garlic is a potent antimicrobial substance. In the present study, we aimed at understanding the molecular mechanism of its antibacterial effects.

We first tested allicin for its ability to inhibit growth of a set of pathogenic organisms. Growth of the microorganisms was inhibited by allicin concentrations ranging between 32 and 64 μg ml^−1^. A remarkable difference in the effective concentration of allicin was found for the *P. aeruginosa* DSM50071 strain tested. The unusually high concentration of 512 μg ml^−1^ necessary to prevent growth has been reported before for this organism (44) and suggests that concentrations of allicin needed to be effective against some strains of this organism are therapeutically not achievable. In minimal medium, allicin exhibited an MIC of 23 μg ml^−1^ against *E. coli* MG1655. These results are in good agreement with a previously reported LD_50_ of 15 μg ml^−1^ allicin against *E. coli* (45). To achieve inhibition of growth in exponentially growing *E. coli*, we needed to add substantially higher concentrations. Given that the main target of allicin are thiols, which occur in high abundance (29 mm) in the *E. coli* cell (Fig. 2*A*), the substantially higher number of cells present in a midlogarithmic culture (∼10^8^ cells ml^−1^) *versus* the small inoculum used for MIC tests (2 × 10^4^ cells ml^−1^) could account for this difference.

After treatment of *E. coli* with a sublethal amount of allicin, total cellular sulfhydryl levels dropped significantly, which seems to be a cumulative effect of the conversion of GSH to its *S*-allylmercapto adduct (Fig. 4) and modification of protein cysteine thiols (Fig. 7). Interestingly, a one-time submillimolar dose of allicin causes the cellular thiol content to drop by 11 mm, indicating that allicin permeates the cell much faster than the cellular repair system can remove the thiol modifications caused by it. GSH is one of the most important cellular antioxidants and together with its oxidized counterpart GSSG constitutes the main thiol redox buffer. We could show that the loss in free thiols is caused, at least in part, by modification of this tripeptide to *S*-allylmercaptoglutathione, a known reaction product of GSH with allicin *in vitro* (16). Glutathione oxidoreductase seems then unable to return *S*-allylmercapto-modified glutathione to the cellular GSH pool.

Loss of reduced GSH *in vivo* could result in oxidative stress. Such a drop in free GSH caused by allicin was also shown for *S. cerevisiae* with the help of genetically encoded, fluorescent reduction-oxidation-sensitive GFP probes (18). At the concentrations used in *S. cerevisiae*, this drop coincided with induction of apoptosis in a proportion of the cells. In contrast, in our experiment, when *E. coli* cells were treated with 128 μg ml^−1^, they resumed growth after ∼90 min of growth arrest. The cultures reached the same final optical densities when compared with the control, indicating that effective mechanisms to overcome allicin stress exist in *E. coli*. Indeed, lowering of the GSSG levels compensates for the loss in total GSH in allicin-treated *E. coli*. This results in a slightly lower GSH/GSSG redox potential (−270 mV) in these cells when compared with wild type (−257 mV) and could be part of the defense strategy of the cell.

Two-dimensional PAGE analysis of the protein expression pattern during treatment with allicin demonstrated that proteins belonging to the heat shock and oxidative stress responses are up-regulated significantly. A similar observation was made previously in our laboratory when cells were subjected to chemically or genetically induced disulfide stress (26). Under these conditions, OxyR is activated by thiol oxidation, and non-native disulfide bonds lead to protein unfolding. The unfolded proteins are then bound by DnaK, which is no longer available to target the heat shock σ factor σ^32^ to proteases for its immediate degradation. σ^32^ is thus stabilized and induces the heat shock response. Although we have shown that allicin, in contrast to diamide, does not primarily induce protein disulfide bond formation in the cytoplasm, allicin exposure resulted in enhanced protein aggregation accompanied by increased stability of σ^32^. This finding thus could explain the observed induction of heat shock proteins.

Many up-regulated proteins showed a shift in pI on our two-dimensional gels, indicating a direct modification by allicin. Mass spectrometric analysis of lysates in a gel-free approach showed that, 15 min after allicin treatment, a substantial set of proteins is *S*-allylmercapto-modified. To our knowledge, a global study that analyzes the total proteome of an organism for allicin-induced modifications has not been reported yet. Currently, a study of the allicin yeast redoxome is in progress in our laboratories, and it will be interesting to compare the response in yeast with that in *E. coli*. Recently, a *Burkholderia cepacia* peroxiredoxin, purified after expression in *E. coli* was shown to be *S*-allylmercapto-modified after treatment with allicin *in vitro*, as determined by mass spectrometry (15). The *S*-allylmercapto-modified proteins identified in our study are mostly high abundance proteins of primary metabolism, indicating that allicin modifies proteins unspecifically. Interestingly, none of the peptides detected for GapA, GrxC, or AceA contained catalytically relevant or already described redox-sensitive cysteines. OxICAT analysis of these described redox-active cysteines revealed no significant change in their oxidation state in GapA and GrxC. We were able to quantify the redox state of cysteine 195 in AceA (isocitrate lyase); this cysteine residue is adjacent to the substrate binding site (42) in two of our three biological replicates. The oxidation state of Cys-195 changed by only 18 percentage points as opposed to 51 for Cys-244, suggesting that in isocitrate lyase, too, an allosteric cysteine is the major target of allicin.

AceA was tested for its activity *in vivo*. Allicin exposure of cells significantly reduced their isocitrate lyase activity. A number of enzymes from both pro- and eukaryotic organisms have been reported to be inhibited by allicin *in vitro* (14, 46). Rabinkov *et al.* (16) reported that papain and alcohol dehydrogenase were efficiently inactivated by allicin as well as by GSSA *in vitro*. In papain, Cys-25 is part of the active site, whereas in alcohol dehydrogenase Cys-203 is located close to the NADP^+^ binding site. Activity of both enzymes could be restored by DTT or 2-mercaptoethanol, and a released allyl mercaptan moiety was detected. In the present study, we could exemplarily demonstrate that enzyme inhibition *in vivo* coincides with the *S*-allylmercapto modification of cysteines in cells exposed to allicin. Although only about half of Cys-244 of AceA was found to be modified *in vivo*, activity dropped by significantly more than 50%. This points to an allosteric effect where the modification of one subunit might cause the inactivation of the isocitrate lyase tetramer or an additive effect of allosteric Cys-244 and active site Cys-195 modification.

Taken together, it seems likely that accessible cysteines that are part of the active sites of the enzymes can be reduced by the major reducing systems *in vivo* and thus appear mostly reduced in our approach. In contrast, cysteines that are buried in the core of a protein might still be accessible to the small hydrophobic molecule allicin but potentially cannot be reached by the repair machinery. Indeed, most cysteines affected by *S*-allylmercapto modification in our study had an accessible surface area well below 25%, which is a threshold above which an amino acid could be considered surface-accessible (47) (Table 2).

Overall, the mechanism of action of allicin is thus likely a combination of the decrease of reduced glutathione levels through the formation of GSSA and inhibition of enzymes crucial for the metabolism of the cell and the oxidative stress defense through *S*-allylmercapto modification. This modification could be formed directly by reaction of cysteine residues with allicin or through the reaction of GSSA with protein cysteines (16).

At sublethal allicin concentrations, cells can overcome stress triggered by allicin. The inability of an *rpoH*-deficient *E. coli* strain to resume growth after allicin exposure demonstrates that the induction of the heat stress response is essential for this recovery.

When compared with most clinically applied antibiotics, allicin seems to have an advantage. It does not target a specific protein so that resistance development by target mutation is therefore rather unlikely. However, its high instability in the gastric tract and therefore inability to reach the desired target and its high toxicity when subcutaneously injected prevent its systemic clinical use as an antibiotic (48–50). Furthermore, the high GSH concentration in mammalian tissues and blood is likely to make it difficult to achieve therapeutically relevant concentrations of allicin in the body. Additionally, the targets we identified in *E. coli* are not specific to bacteria but essentially occur in all living beings and thus will be attacked in human cells as well (51). Nevertheless, because allicin is volatile, direct inhalation of allicin vapor or aerosols may be able to target pulmonary infections. This promising line of investigation is being pursued in our laboratories.

Along the same lines, first steps have been made for the site-specific application of allicin. In 2010, a monoclonal antibody against *Aspergillus fumigatus* was fused to alliinase (52). *In vivo* application of this antibody together with the substrate of the enzyme, alliin, successfully cleared intratracheal fungi in mice. These efforts together with a deeper understanding of the mode of action of allicin make this molecule a promising candidate structure for the treatment of numerous human diseases.

## Author Contributions

A. M. performed glutathione determinations, crude extract aggregation assays, RpoH stability assays, growth tests with RpoH mutant, analysis of the experimental data, and wrote the manuscript. J. E. performed growth tests; MIC determination of *E. coli*; assays for sulfhydryl contents, alkaline phosphatase activity, and isocitrate lyase activity; prepared mass spectrometry samples, and drafted parts of the manuscript. Two-dimensional PAGE, protein identification, and MIC determination of microorganisms were carried out by P. P. Analysis of two-dimensional gels and analysis of data for protein identification was done by J. E. B. Allicin was synthesized by F. A. F. A. and A. J. S. contributed to editing and discussion of the manuscript. Mass spectrometry for *S*-allylmercapto-modified protein identification and OxICAT experiments were performed by K. K. L. I. O. L. conceived the experiments and wrote part of the manuscript.

## Supplementary Material

Supplemental Data

## Footnotes

5 The abbreviations used are:

MIC: minimal inhibitory concentration
DTNB: 5,5′-dithiobis(2-nitrobenzoic acid)
ICAT: isotope-coded affinity tag
MS/MS: tandem mass spectrometry
TNB: thio-2-nitrobenzoic acid
OxICAT: isotope-coded affinity tag labeling to determine the oxidation state of thiols
Bis-Tris: 2-[bis(2-hydroxyethyl)amino]-2-(hydroxymethyl)propane-1,3-diol
GSSA: *S*-allylmercaptoglutathione.
