# Supplementary material for: Allicin Induces Thiol Stress in Bacteria through S-Allylmercapto Modification of Protein Cysteines
Source: J Biol Chem. 2016 Mar 23;291(22):11477–90. doi: 10.1074/jbc.M115.702308 (PMC4882420; doi:10.1074/jbc.M115.702308)
Supplement: Supplemental Data [file supp_291_22_11477__index.html]

Allicin Induces Thiol Stress in Bacteria through S-allylmercapto Modification of Protein Cysteines — Allicin Induces Thiol Stress in Bacteria through S-Allylmercapto Modification of Protein Cysteines — Allicin-modified Proteins in E. coli — Supplemental Data 

# Allicin Induces Thiol Stress in Bacteria through *S*-Allylmercapto Modification of Protein Cysteines

## Supplemental Data

- Supplemental Table 1 (.xlsx, 38 KB) - Details about the mass spectrometric identification of protein spots from 2D-PAGE gels (additional information to Table 1 and Figure 3 of the main text).
- Supplemental Table 2 (.xlsx, 85 KB) - Mass spectrometric identification data of S-allylmercapto-modified peptides.
- Supplemental Table 3 (.xlsx, 92 KB) - S-allylmercapto-modified peptides quantified by OxICAT (sheet "all\_quantified"). In the sheet "TOP12", the 12 peptides with a percentage change in modification above 10?% are displayed (see also Table 2).
